# The Distribution of Subjects in L2 Spanish by Greek Learners

**DOI:** 10.3389/fpsyg.2021.794587

**Published:** 2022-01-20

**Authors:** Panagiota Margaza, Anna Gavarró

**Affiliations:** Departament de Filologia Catalana, Universitat Autònoma de Barcelona, Barcelona, Spain

**Keywords:** null subjects, preverbal subjects, postverbal subjects, syntax-pragmatics interface, syntax-semantics interface, Interface Hypothesis, L1 Greek – L2 Spanish

## Abstract

Our study examines the expression and position of subjects in L2 acquisition, two phenomena that are studied within the framework of the Interface Hypothesis (IH). The first version of the IH predicts that interface properties involving syntax and another cognitive domain may not be fully acquirable in a second language (Sorace and Filiaci, 2006; also Sorace, 2011). The second version of the IH predicts that formal properties involving the syntax-semantics interface are unproblematic to acquire in L2 grammars compared to the vulnerable properties integrating syntax with the higher level of pragmatics (Tsimpli and Sorace, 2006). We test these IH versions in L2 Spanish as acquired by L1 Greek speakers, a language combination understudied in the literature. Both languages share the null subject parameter, but still the IHs predict incomplete command at the syntax-pragmatics interface. Two acceptability judgment tasks were designed for Spanish: the first task tested null/overt subjects in referential contexts and the second task tested preverbal/postverbal subjects in informational contexts. Participants were L1 Greek intermediate and advanced learners of Spanish and native speakers of Spanish (15 subjects in each group). In the first task, both experimental groups showed target-like distribution of null/overt subjects in most non-contrastive and contrastive contexts, except for the advanced group in unambiguous referential contexts. In the second task, the respective groups accepted felicitous preverbal subjects with unergative verbs, but diverged from native-like distribution of postverbal subjects with unaccusative verbs in neutral contexts. The L2 groups showed a high preference for unfelicitous preverbal subjects with both intransitive verbs in informational contexts, contrary to the subject inversion patterns of the control group. The results obtained were not consistent with the IH predictions, and other factors such as the type of subject, verb class and context played a role in L2 performance.

## Introduction

In this study, we explore the acceptance of null/overt subjects and preverbal/postverbal subjects in specific pragmatic contexts in L2 Spanish acquisition by Greek learners. Our aim is to examine both the expression and position of subjects within the framework of the Interface Hypothesis (IH), as most studies examine either the expression of null/overt subjects (see Clements and Domínguez, 2017; Lozano, 2018) or the position of preverbal/postverbal subjects (see Lozano, 2006a,b; Domínguez and Arche, 2014); scarce previous work on L2 Spanish addresses these two properties of the null subject languages. After studying the anaphora resolution of null/overt subjects, Sorace and Filiaci (2006) introduced the first version of the IH (IH-1 hereafter), which predicts that interface structures involving the mappings between syntax and other cognitive domains such as pragmatics are more complex to acquire in L2. After examining focalization and subject uses, Tsimpli and Sorace (2006) proposed a new version of the IH (IH-2 hereafter), arguing that structures involving the syntax-semantics interface are easier to acquire than structures involving the syntax-pragmatics interface. In this study, we aim at testing these versions of the IH in an understudied language combination, L1 Greek – L2 Spanish, Greek and Spanish being two languages that share the null subject value and the unergative/unaccusative universal distinction. Still, the two languages present some differences in the position of subjects in informational focus contexts. In this case, our main aim is to examine if acquiring interface phenomena remains difficult given the similar distribution of subjects in these two null subject languages.

This paper is structured as follows: section “Background” presents the IH and examines the distribution of subjects in L2 Spanish. Section “The Study” presents our study: the IH predictions and the methods of our research, including the experimental design, the procedure and the data analysis. The main results of our analysis are detailed in section “Results.” The discussion of the substantial findings and the overall conclusions of our study appear in section “Discussion and Conclusions.”

## Background

### The Interface Hypothesis: Versions and Objections

Sorace and Filiaci (2006) proposed the first version of the Interface Hypothesis, as defined in (1).


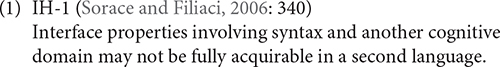


In their work, Sorace and Filiaci (2006) examined anaphora resolution at the syntax-pragmatics interface, concerning the use of null/overt subjects in appropriate contexts. In particular, they focused on the mastery of anaphora resolution in L2 acquisition of Italian by L1 English. In their results, the L2 learners had problems with the interpretation of overt pronouns in relation to their antecedents, but showed target-like processing of null pronouns. This asymmetry between null and overt subjects was not consistent with the IH-1, as both types of subjects were predicted to present target-deviant distribution at the syntax-pragmatics interface.

Tsimpli and Sorace (2006) also proposed a second version of the IH (IH-2 hereafter), taking into consideration the distinction between the syntax-semantics interface, involving the formal properties of grammar and the syntax-pragmatics interface, involving a higher level of language use, integrating properties of language and pragmatic processing. Their definition of the IH is stated in (2).


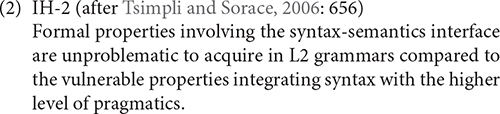


In their study, Tsimpli and Sorace (2006) examined word order in relation to focalization and the expression/omission of subjects in relation to person features (1st/2nd/3rd). The two phenomena were examined based on L1 Russian learners’ performance in L2 Greek. The results showed that the L2 learners had acquired the felicitous word order with focalization, while they overused 1st/2nd against 3rd person overt pronouns, showing that person had an effect on L2 performance. The authors claimed that word order at the syntax-semantics interface was easier to acquire than subject use when the syntax-pragmatics interface was involved, in support of the IH-2. However, in their own results L2 learners had no problems with the distribution of null subjects, so that the syntactic-pragmatic constraints were not always compromised.^1^

Sorace (2011: 15) rephrased the IH as follows: “L2 learners are less efficient than monolinguals at processing structures at the syntax-pragmatics interface because their knowledge of or access to computational constraints is less detailed or less automatic than in monolinguals and they have fewer cognitive resources to deploy on the integration of different types of information in real-time language use.” The syntax-pragmatics interface is claimed to be the main locus of processing difficulties and acquisition delays at the highest levels of L2 ultimate attainment. Interface problems are attributed to the fact that L2 learners need to acquire both the representational knowledge of the structure and the mapping conditions that operate within interface components, and the processing principles that apply in real-time integration of different domains. Sorace (2012: 210) explicitly states that there is “a hierarchy of computational difficulty” with structures requiring proceduralized “internal” mappings being less taxing than structures requiring the integration of contextual information.

White (2011b: 109) questioned Sorace’s claim that the IH does not hold at all L2 developmental stages, since interface problems, should they occur, would not emerge out of the blue, but appear in the course of L2 language development, not only at the near-native stages. White (2011a: 588) also argues that even if L2 non-native performance reveals processing difficulties in acquiring interface phenomena, this does not imply permanent impairment at the interfaces. Slabakova (2009) and White (2011a,b) also cast doubt on what is considered “difficult” and what “easy” under the IH, as the syntax-pragmatics interface is not necessarily found to be more problematic than other linguistic domains, such as the syntax-semantics interface (see for example Serratrice et al., 2009; Sorace et al., 2009). The argument is therefore that it is inappropriate to make broad generalizations for interface domains.

Taking into consideration that not all syntax-discourse interface properties are equally problematic, Lozano (2016) formulated a more specific proposal, the Pragmatic Principles Violation Hypothesis (PPVH), which makes predictions mostly on the distribution of null/overt subjects. In his study, Lozano (2016) examined anaphora resolution in L2 Spanish by L1 English speakers. His results showed that the L2 learners presented native-like use of null subjects in topic-continuity contexts, while they avoided this type of subject in topic-shift contexts. Regarding overt pronouns, the L2 learners preferred the expression of pronominal subjects in topic-shift contexts, but also used this type of subject in topic-continuity contexts. Thus, it was easier to overuse the unfelicitous overt pronoun in topic-continuity contexts than to resort to the unfelicitous null pronoun in topic-shift contexts. These results were against the predictions of the IH of complete vulnerability at the syntax-pragmatics interface. Based on pragmatic principles related to redundancy and ambiguity, following the neo-Gricean principles of Informativeness and Manner, he proposed that overt-when-null violation to mark topic-continuity leads only to redundancy and not to informative breakdown, while null-when-overt violation to mark topic-shift leads to ambiguity that causes communicative failure in discourse. He stated his hypothesis as in (3).


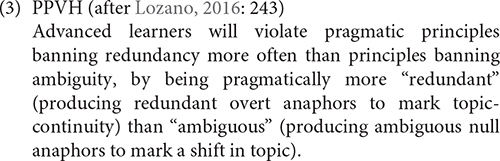


This proposal follows the IH in placing the syntax-pragmatics interface as the locus of delay in L2 acquisition, but is more restrictive.

### Subject Distribution in L2 Spanish

Spanish and Greek share the null subject parameter (4) (see Fernández-Soriano, 1989 for Spanish and Philippaki-Warburton, 1987, 1989 for Greek) and the unergative/unaccusative distinction affects word order (5) and (6) (see Eguren and Fernández-Soriano, 2004 for Spanish and Alexiadou and Anagnostopoulou, 2004 for Greek). However, the two languages differ with respect to subject position in informational contexts, VS in Spanish and SV in Greek (see Roussou and Tsimpli, 2006), as illustrated in (7) and (8).


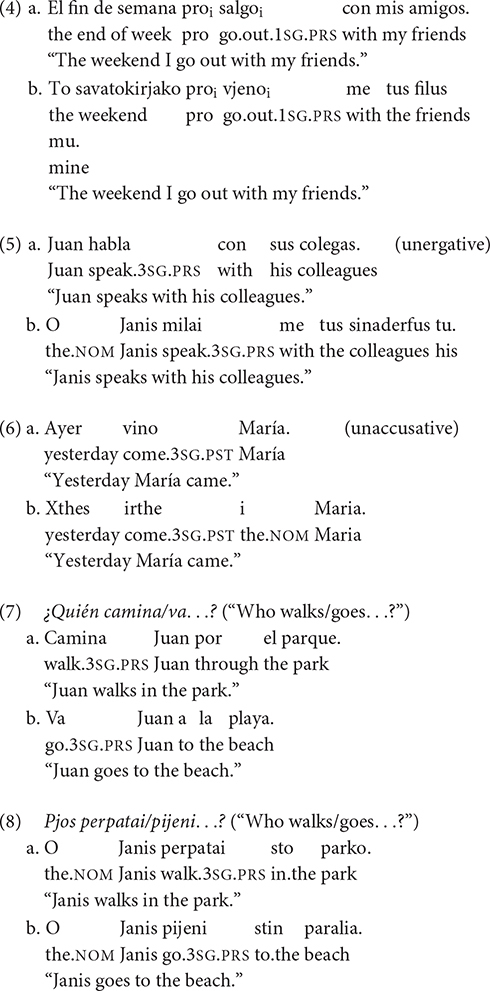


While L2 Spanish acquisition with regard to subject expression and subject position is common in the literature, the combination of L1 Greek and L2 Spanish is understudied. Lozano (2018) explored the distribution of null/overt subjects in L2 Spanish by L1 Greek learners. He focused on the development of pronominal subjects at three proficiency levels (intermediate, lower-advanced, and upper-advanced). In contrastive contexts, all L2 groups distinguished the felicitous overt pronoun from the unfelicitous null pronoun. In this case, the upper-advanced group showed convergence with native behavior, but presented some persistent deficits in topic-continuity contexts, in which they accepted redundant overt pronouns. At lower levels, L2 learners alternated between null and overt subjects, confirming a higher divergence from native-like patterns. Lozano (2009) also claimed that deficits at the syntax-pragmatics interface were selective, as the type of person played a role in the performance of learners who had problems with the anaphoric uses of 3rd person pronouns, while they presented better mastery of the deictic uses of 1st/2nd person. Lozano (2006a) examined subject-verb alternations in the data of three (upper-intermediate, lower-advanced, and upper-advanced) experimental groups of L1 Greek learners of L2 Spanish. All L2 groups showed native-like mastery of the felicitous subject position with intransitive verbs at the syntax-semantics interface. On the other hand, all L2 groups had problems with the distribution of subject-verb at the syntax-pragmatics interface, except for the upper-advanced group that showed a clear preference for the felicitous VS with unaccusative verbs in informational contexts. Lozano’s (2006a) results supported again native-like command of syntactic-semantic properties, but did not confirm that all syntactic-pragmatic properties were inacquirable due to permanent vulnerability. Hertel (2003) also found that learners of higher levels performed native-like at the syntax-pragmatics interface, so that discourse-related word order was eventually acquired in L2.

Domínguez and colleagues also examined subject distribution in L2 Spanish by L1 English speakers. Clements and Domínguez (2017) found that, in contrastive contexts, L2 learners at an advanced level followed native-like intuitions with respect to the use of overt subjects, while they performed target-deviant in the case of unfelicitous null subjects. In switch referent contexts, though, advanced learners did not differ from native-like performance when the felicitous option was a null pronoun, showing command of the pragmatic constraint involving the possibility of omitting subjects in topic-shift contexts. In non-topic-shift contexts, L2 learners also approached the rates of natives, showing no problems with the use of null pronouns. However, advanced learners did not reject unfelicitous overt pronouns to the same extent as the control group. The problematic nature of the syntax-pragmatics interface was not always supported by the results. Domínguez and Arche (2014) also examined the position of subjects at three proficiency levels (beginner, intermediate, and advanced). Results indicated that beginner and intermediate groups preferred SV in all contexts, while the advanced group accepted VS over SV with unaccusative verbs in broad and narrow focus contexts, but showed optionality between the two word orders with unergative verbs in focus contexts. Subject inversion acquisition was a slow process, as systematic preference for inversion was only observed at advanced levels. Persistent SV/VS problems were caused not exclusively by the syntax-pragmatics interface, but also by the syntax-semantics interface when verb class was involved (see also Montrul, 2005).

## The Study

### Predictions

In this study, we also examine the expression and position of subjects in various pragmatic contexts in Spanish. The novelty of our study is that we give an account of both phenomena, as in the literature most studies examine either the use of overt/null subjects (Clements and Domínguez, 2017; Lozano, 2018) or the position of preverbal/postverbal subjects (Lozano, 2006a,b; Domínguez and Arche, 2014). The combination of two null subject languages, L1 Greek and L2 Spanish, is also new, as in most studies the combination involves a non-null subject language and a null subject language, e.g., L1 English and L2 Spanish (see Hertel, 2003; Montrul, 2005; Rothman, 2009). Our main aim is to examine the extent to which Greek learners of Spanish show command of both null/overt subjects and preverbal/postverbal subjects in referential and informational contexts. These phenomena are tested under the light of the two versions of the IH as well as Lozano’s (2016) PPVH. The predictions of the above hypotheses are as follows.

i According to the IH-1, L2 learners are expected to accept the unfelicitous type of subjects, null or overt, preverbal or postverbal in non-contrastive, unambiguous and contrastive referent-shift contexts as well as informational contexts in which the syntax-pragmatics interface is involved.^2^
ii According to the IH-2, L2 learners are expected to accept the felicitous subject position with intransitive verbs in neutral contexts in which the syntax-semantics interface is involved, while they will have problems with subject expression and position in referential and informational contexts in which the syntax-pragmatics interface is involved.
iii If Lozano’s (2016) PPVH is accurate, L2 learners will overuse overt pronouns in referential contexts in which a null pronoun is expected, while they will perform target-like in pragmatic contexts in which an overt pronoun is expected. Pragmatic failure leading to redundancy is predicted but not leading to ambiguity.

### Methods

In our study, we apply offline tasks that examine subject processing under a particular time limit in the discourse. We have chosen a written task that allows to better control subject acceptability in relation to the type of context, referent antecedent and verb class. Contextualized pragmatic felicitousness judgment tasks have been widely used in applied studies on L2 Spanish acquisition (see Hertel, 2003; Lozano, 2006a,b, 2018; Clements and Domínguez, 2017), so this is a suitable method to examine the real preferences of L2 learners.

#### Materials: Experimental Design

We designed two acceptability judgment tasks to examine the type of subject, null or overt, preverbal or postverbal in contextualized sentences in Spanish. These experiments include a 5-point Likert-scale from −2 (fully rejected), −1 (rejected), 0 (neither rejected/neither accepted) to 1 (accepted) and 2 (fully accepted); this allows the rating of the exact degree of acceptability of the type of subjects in various pragmatic contexts (see also Lozano, 2006a,b, 2018).

Experiment 1 consists of a total number of 21 stimuli: 16 items with two sentences each (total: 32 sentences) testing the acceptance or rejection of null/overt subjects and five distractors (total: 10 sentences). The variables checked are: (i) person (1st, 2nd, 3rd) and (ii) discourse context (referent-continuity, unambiguous referent-shift, and contrastive referent-shift). Experiment 2 consists of a total number of 25 stimuli: 20 items with two sentences each (total: 40 sentences) testing the acceptance or rejection of preverbal/postverbal subjects and five distractors (total: 10 sentences) that do not involve the phenomena examined, so that they are not further analyzed. The variables controlled for are: (i) verb class (unergative and unaccusative) and (ii) context (neutral or informational).

Experiment 1 includes three subtest conditions. Subtest 1 involves the acceptability of 1st and 2nd person null/overt subjects in non-contrastive referential contexts. This subtest contains six items: three items with 1st person subjects and three items with 2nd person subjects. Both types of person demand the production of null subjects in non-contrastive referential contexts. The variables tested are *Person* and *Subject* type in a given context, giving rise to the following conditions: (i) 1st person, null subject, non-contrastive referential context, (ii) 1st person, overt subject, non-contrastive referential context, (iii) 2nd person, null subject, non-contrastive referential context, and (iv) 2nd person, overt subject, non-contrastive referential context. Under the IH-1 and IH-2, L2ers are expected to accept unfelicitous overt pronouns of both persons due to the involvement of pragmatics; likewise, under Lozano’s (2016) PPVH, L2ers will overaccept overt pronouns when null pronouns are expected.

To illustrate, in example (9) the null subject is felicitous in referent-continuity contexts in which the inflection of the verb *volver* (“return”) shows the 1st person singular in (9a). In this case, the expression of the overt pronoun *yo* (“I”) (9b) would be redundant. Still, an overt pronoun would be acceptable with emphatic/contrastive interpretation, but this is not the first choice in the sentence examined in Spanish. (In Greek, subject omission is also the preferred option in the equivalent contexts).


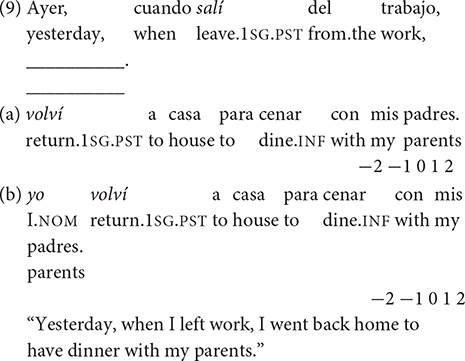


Subtests 2 and 3 involve the acceptability of 3rd person null/overt subjects. Subtest 2 consists of five items requiring null subjects in referent-shift contexts (with one unambiguous antecedent). The variables tested are 3rd *Person* and *Subject* type in a given context, giving rise to the following conditions: (i) 3rd person, null subject, referent-shift context (with one antecedent), and (ii) 3rd person, overt subject, referent-shift context (with one antecedent). Since the distribution of null pronouns is constrained by the syntax-pragmatics interface, the IH-1 and IH-2 predict that L2ers will fail to accept the felicitous type of pronoun in unambiguous referent-shift contexts; similarly, Lozano’s (2016) PPVH predicts that L2ers will be target-deviant, as in the previous subset of items.

In example (10) the inflection of the verb *decir* (“say”) allows the identification of the 3rd person of the antecedent referent *el profesor* (“the teacher”) so that the production of a null subject is acceptable in Spanish (10a). However, an emphatic/contrastive overt pronominal subject *él* (“he”) is possible in referent-shift contexts (10b). (In Greek, a null subject is also the first choice in the equivalent contexts, but the expression of an overt pronoun is not disallowed).


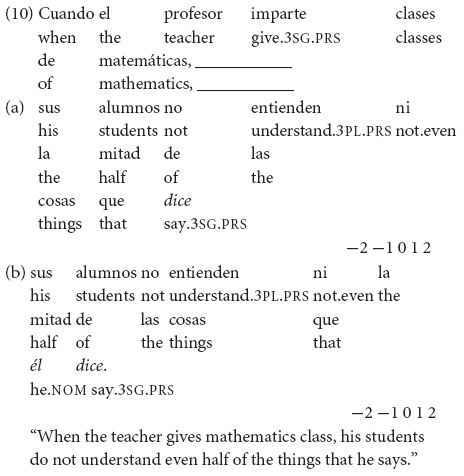


Subtest 3 involves five items that require the expression of 3rd person subjects. The variables tested are 3rd *Person* and *Subject* type in a given context, giving rise to the following conditions: (i) 3rd person, overt subject, contrastive referent-shift context (with two antecedents), and (ii) 3rd person, null subject, contrastive referent-shift context (with two antecedents). Under the IH-1 and IH-2, L2ers will fail with the felicitous overt pronoun, due to difficulties in acquiring referential properties. On the other hand, Lozano’s (2016) PPVH predicts that L2ers will perform target-like.

In example (11), the inflection of the verb *hablar* (“speak”) shows the 3rd person singular in Spanish, but it does not distinguish the antecedent referents *Manolo* or *Sofía*, so the expression of the pronoun *él* (“he”) is obligatory in (11a) to refer to the antecedent (*Manolo*). A null subject would generally refer to the closest singular antecedent (*Sofía*) in the discourse (11b). (In Greek, the felicitous option is also an overt pronoun in the equivalent contexts).


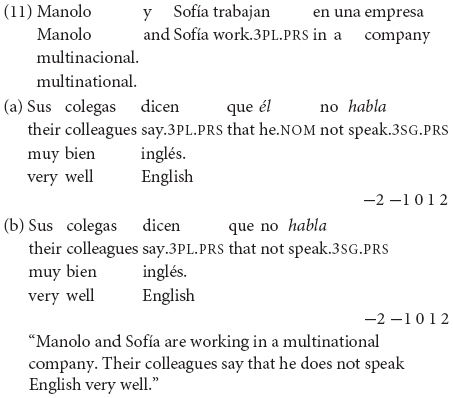


In Experiment 2, we examine the position of subjects with intransitive verbs in various contexts. Two subtest conditions are included. Each subtest contains five items with unergatives and five with unaccusatives. Subtest 1 involves the unergative/unaccusative distinction that allows the anteposition of unergative subjects and the postposition of unaccusative subjects (see also Lozano, 2006a,b). This distinction is examined in direct question-answer pairs in which the informational focus is neutral, so the syntactic-lexical-semantic properties of verbs constrain the position of their subjects. The variables tested are *Word Order* and *Verb Class* in a given context, giving rise to the following conditions: (i) SV, unergative verb, neutral context, (ii) VS, unergative verb, neutral context, (iii) SV, unaccusative verb, neutral context, and (iv) VS, unaccusative verb, neutral context. Under the IH-2, L2 learners will accept the felicitous subject position with both verb classes at the syntax-semantics interface.

In the contextualized examples (12) and (13), the broad focus questions *>Qué sucede?* (“What happens?”) and *>Qué sucedió en el banco?* (“What happened in the bank?”) trigger as new information the entire answer, allowing SV with the unergative *caminar* (“walk”) in (12a) and VS with the unaccusative *entrar* (“enter”) in (13a). On the other hand, the second word order option is not acceptable in Spanish neutral contexts in (12b) and (13b). (In Greek, unergative verbs also accept the SV order, while unaccusative verbs allow the VS order in neutral contexts).


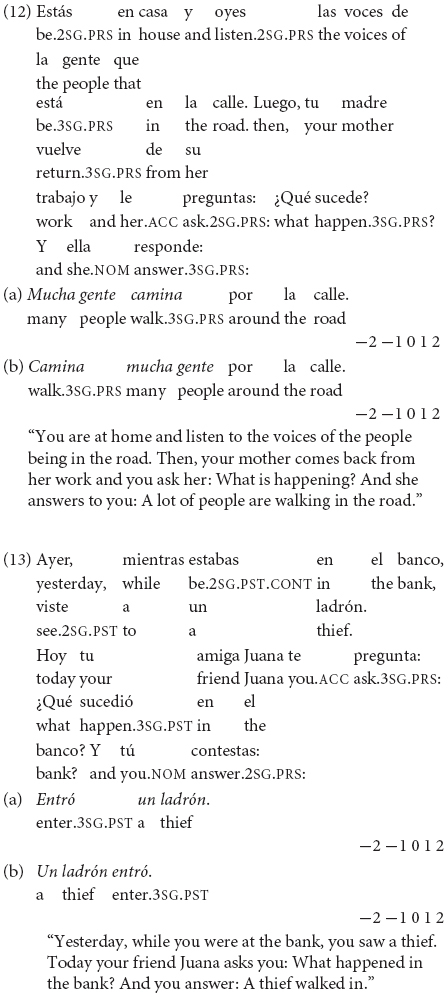


Subtest 2 examines the distribution of subjects in informational focus contexts. In this case, the syntactic-pragmatic properties of focus determine word order, so the felicitous word order is VS with both unergative/unaccusative verbs. The examined contexts contain direct question-answer pairs, as in Subtest 1, but, in this case, the question is with *>Quién*…*?* (“Who.?”), triggering a focalized subject that introduces new information into the discourse. The variables tested are also *Word Order* and *Verb Class* in a given context, giving rise to the following conditions: (i) SV, unergative verb, informational context, (ii) VS, unergative verb, informational context, (iii) SV, unaccusative verb, informational context, and (iv) VS, unaccusative verb, informational context. Under both the IH-1 and IH-2, L2ers will accept the unfelicitous option of preverbal subjects as the syntax-pragmatics interface is involved in the contexts examined. Lozano’s (2016) PPVH does not make predictions for this subset of cases.

In the contextualized examples (14) and (15), the narrow focus question with *>Quién.?* (“Who…?”) receives as answer the VS order with the unergative *reírse* (“laugh”) in (14a) and the unaccusative *venir* (“come”) in (15a). The anteposition of subjects is unacceptable in (14b) and (15b) contexts in Spanish. (In Greek, though, the felicitous option is the SV order with both intransitive verbs in informational focus contexts).


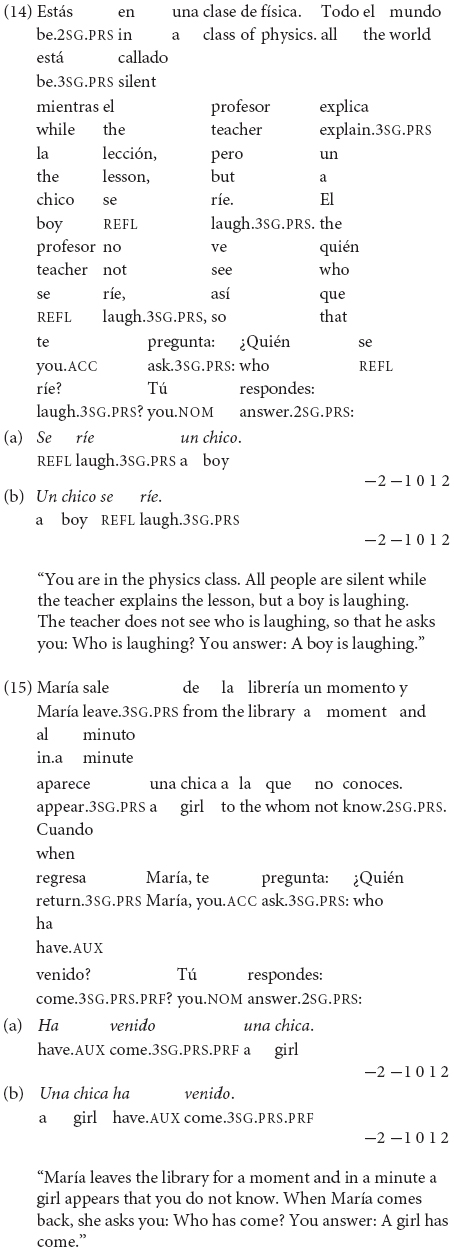


All test items were fully randomized.^3^

#### Participants

Participants of both experiments were two groups of L1 Greek learners of L2 Spanish and a group of native Spanish speakers. The non-native groups consisted of intermediate and advanced students who were learning Spanish as an L2 at the Instituto Cervantes de Atenas. At the time of the experiments, intermediate and advanced learners were attending the respective third and fifth Spanish courses for 4 hours a week. Both groups had passed the Examination for the Diploma of Spanish as a Foreign Language (*DELE)*. Intermediate learners had obtained an average rate of 86% in the B1 Exam, while advanced learners had attained a mean of 89% in the C1 Exam, according to the European Framework for Foreign Languages. Spanish native speakers were living in Madrid and were students at the Universidad Autónoma de Madrid. This third group served as a control group and established the rate of acceptability of the various types of subjects in Spanish. Table 1 shows the essential information for the three groups.

**TABLE 1:** Participants.

Groups	Intermediate	Advanced	Control
First language	Greek	Greek	Spanish
Number	15 (3 males and 12 females)	15 (2 males and 13 females)	15 (5 males and 10 females)
Age range (SD)	30-60 (9.27)	34-62 (8.24)	30-50 (7.07)
Studies in L2 Spanish	3rd L2 course	5th L2 course	——
Duration	3 years	5 years	——
Proficiency level	B1	C1	Native
Average score in *DELE* exams	86%	89%	——

#### Procedure

Both Experiments 1 and 2 were administered in the Instituto Cervantes de Atenas, where the classes of L2 Spanish were taught and in the Universidad Autónoma de Madrid, where the native speakers were studying. All participants provided written informed consent to participate in the tasks of our study. Participants also answered a language questionnaire about their L1 (Greek being the only L1 examined in this study), and their knowledge of L2 Spanish, to distinguish between two competence levels for L2 learners, which determined the factor of Group in the statistical analysis. Then all groups were instructed as to how to complete the acceptability judgment tasks and how to rate the two-sentence items. The five points of the scale were explained, as follows: −2 (fully rejected), −1 (rejected), 0 (neither rejected nor accepted), 1 (accepted), and 2 (fully accepted). The participants were also given a distractor example that indicated how to rate the felicitous and unfelicitous options. All questions and doubts were answered to avoid misunderstandings. The duration of each task was 45 min, but participants were given extra time if necessary.

#### Coding of Data and Statistical Analysis

In Experiments 1 and 2, the ratings of subject types (null/overt or preverbal/postverbal) on the five-point scale were classified as follows: the accepted (1, 2) and rejected (−1, −2) values were grouped together, while the neither accepted/rejected (0) value was also noted as third category. Subjects were coded in accordance with the context of each given condition. For each condition, a Generalized Linear Model (GLM) was used to compare percentages of accepted items (1, 2) across different levels, using the binomial distribution (see Dobson and Barnett, 2018). Also we examined the interaction of *Person* (1st and 2nd) and *Group* (intermediate, advanced, and control) in Experiment 1 and *Verb Class* (unergative and unaccusative) and *Group* in Experiment 2 applying a Generalized Linear Mixed Model (GLMM) of accepted items (1, 2) with the binomial distribution (see Moscatelli et al., 2012). The GLMM has a high statistical power as it estimates the variability of fixed and random effects. *P*-values were adjusted according to Tukey correction for multiple comparisons. The statistical analysis was performed using the software SAS v9.4, SAS Institute Inc., Cary, NC, United States. The statistical decisions were made taking as significance level the value 0.05.

## Results

Experiment 1 yielded a total of 1,440 responses (480 from each group), while Experiment 2 yielded a total of 1,800 responses (600 from each group). The responses to the distractors were not included in the analysis because they did not involve the phenomena examined. Prior to analysis, the responses were categorized following the grouping of accepted and rejected values, while the zero value was not selected by any of the participants.

Regarding Experiment 1, the number and percentage of accepted values (1, 2) by *Context* (non-contrastive, unambiguous referential, and contrastive) and *Subject* (null/overt) are presented in Table 2. In the three subtest conditions, the L2 intermediate and advanced groups showed a higher rate of felicitous than unfelicitous type of subjects, following the patterns of the control group, except for the advanced group in the overt subject condition in 1st person non-contrastive and unambiguous referential contexts.

**TABLE 2:** Overall means and Standard Deviation in the contexts of Experiment 1.

		Acceptance percentage								
		INTERM			ADVAN			CONTR		
		N	Mean	Std	N	Mean	Std	N	Mean	Std
Non-contrastive context	Null	15	97%	9%	15	99%	4%	15	100%	
	#Overt	15	28%	26%	15	42%	33%	15	36%	34%
1st person	Null	15	98%	9%	15	100%		15	100%	
	#Overt	15	18%	25%	15	42%	41%	15	22%	37%
2nd person	Null	15	96%	12%	15	98%	9%	15	100%	
	#Overt	15	38%	35%	15	42%	37%	15	49%	40%
Unambiguous referential context	Null	15	92%	17%	15	88%	13%	15	97%	7%
	#Overt	15	48%	42%	15	60%	28%	15	33%	40%
Contrastive context	#Null	15	11%	15%	15	15%	26%	15	5%	9%
	Overt	15	89%	21%	15	91%	21%	15	97%	7%

In the GLM, no significant differences between groups for both null/overt subject conditions in non-contrastive and contrastive contexts were found. On the other hand, in unambiguous referential contexts there were significant differences in the overt subject condition (*F* = 5.23, *p*-value = 0.0093). The statistical differences were detected in the comparison between advanced and control groups (*t* = 3.23, adj *p*-value = 0.0066, according to Tukey correction).

**FIGURE 1:**
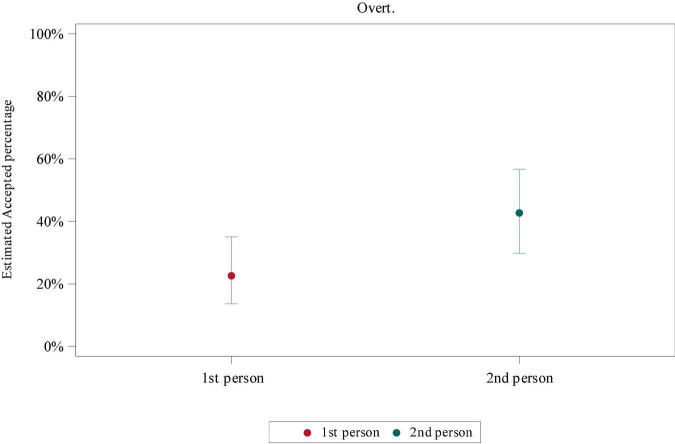
Estimated acceptance percentage according to Person in the overt subject condition.

To examine the interaction of *Person* (1st and 2nd) and *Group* (intermediate, advanced, and control) in non-contrastive contexts, a GLMM was applied. In the null subject condition, there were no significant differences detected. In the overt subject condition there were significant differences with respect to *Person* (*F* = 9.54, *p*-value = 0.0036), but no interaction of *Person* and *Group*. In the *post hoc* test, there were significant differences between 1st and 2nd person (*t* = −3.09, adj *p*-value = 0.0036 according to Tukey correction). See Figure 1 for the 1st vs. 2nd person comparison.

Regarding Experiment 2, the number and percentage of acceptance values (1, 2) by *Context* (neutral and informational), *Verb Class* (unergative and unaccusative), and *Subject* (preverbal/postverbal) appear in Table 3. In the first subtest condition, the L2 intermediate and advanced groups showed a native-like rate of the felicitous preverbal subjects with unergatives in neutral contexts, while both groups showed variability between SV and VS with unaccusatives, diverging from target patterns. In the second subtest condition, both L2 groups showed a higher preference for the unfelicitous subject position in informational contexts, and the intermediate group presented full variability in the case of unaccusatives, against native intuitions for postverbal subjects.

**TABLE 3:** Overall means and Standard Deviation in the contexts of Experiment 2.

			Acceptance percentage								
			INTERM			ADVAN			CONTR		
			N	Mean	Std	N	Mean	Std	N	Mean	Std
Neutral context	Unergative	SV	15	97%	7%	15	95%	21%	15	97%	7%
		#VS	15	37%	28%	15	33%	31%	15	51%	20%
	Unaccusative	#SV	15	76%	31%	15	77%	26%	15	57%	33%
		VS	15	84%	20%	15	79%	22%	15	97%	10%
Informational context	Unergative	#SV	15	85%	18%	15	89%	17%	15	48%	36%
		VS	15	63%	34%	15	55%	33%	15	95%	12%
	Unaccusative	#SV	15	81%	32%	15	87%	25%	15	32%	38%
		VS	15	79%	34%	15	65%	37%	15	97%	10%

In the GLM there were no significant differences between groups in the case of unergatives in the first subtest condition, while the statistical differences were significant in both preverbal (*F* = 4.37, *p*-value = 0.0189) and postverbal subject conditions (*F* = 4.44, *p*-value = 0.0179) with unaccusatives. In the preverbal subject condition, the significant differences were detected in the comparison between advanced and control groups (*t* = 2.58, adj *p*-value = 0.0353, according to Tukey correction). In the postverbal subject condition, the differences were found in both intermediate-control (*t* = 2.48, adj *p*-value = 0.0449, according to Tukey correction) and advanced-control group comparisons (*t* = −2.98, adj *p*-value = 0.0131, according to Tukey correction).

**FIGURE 2:**
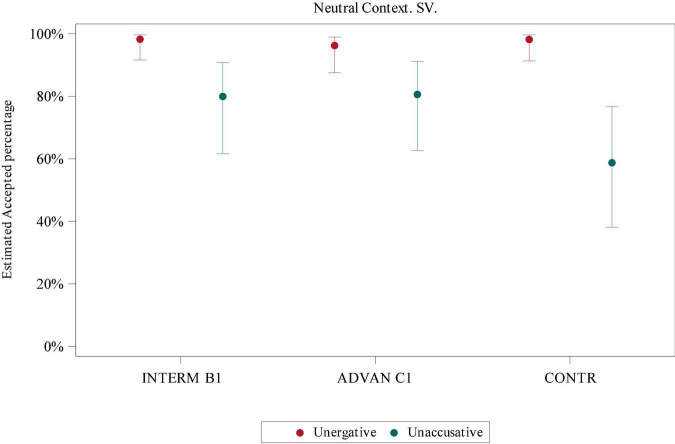
Estimated acceptance percentage for the SV condition in neutral contexts according to Verb Class.

**FIGURE 3:**
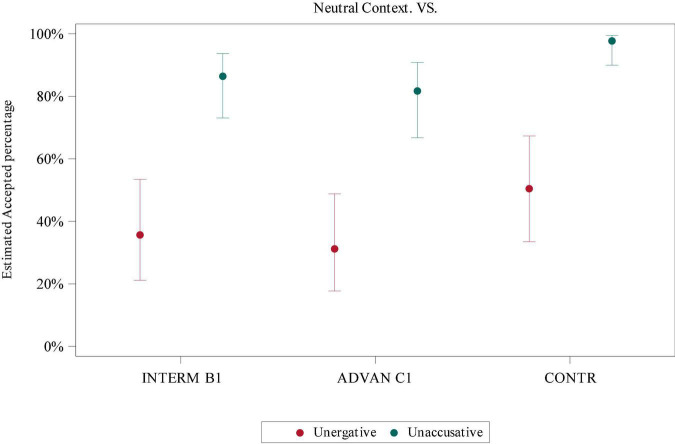
Estimated acceptance percentage for the VS condition in neutral contexts according to Verb Class.

In order to examine the interaction of *Verb Class* (unergative and unaccusative) and *Group* (intermediate, advanced, and control) in neutral contexts, a GLMM was applied. In both SV (*F* = 40.87, *p*-value < 0.0001) and VS conditions (*F* = 75.7, *p*-value < 0.0001), there were significant differences regarding *Verb Class*, but no interaction of *Verb Class* and *Group*. In the *post hoc* test, there were significant differences between unergatives and unaccusatives in both SV (intermediate: *t* = −3.37, adj *p*-value = 0.019; control: *t* = −4.64, adj *p*-value = 0.0005, according to Tukey correction) and VS conditions (intermediate: *t* = 5.69, adj *p*-value < 0.0001; advanced: *t* = 5.58, adj *p*-value < 0.0001; control: *t* = 4.85, adj *p*-value = 0.0002, according to Tukey correction). See Figures 2, 3 for unergative and unaccusative comparison in SV and VS conditions.

In the second subtest condition, the GLM showed that there were significant differences between groups with preverbal (*F* = 17.80, *p*-value < 0.0001) and postverbal subject options (*F* = 11.45, *p*-value = 0.0001) with unergatives in informational contexts. The significant differences were detected in both intermediate-control (preverbal: t: −4.6, adj *p*-value = 0.0001; postverbal: *t* = 4.16, adj *p*-value = 0.0004, according to Tukey correction) and advanced-control group comparisons (preverbal: *t* = 5.02, adj *p*-value < 0.0001; postverbal: *t* = −4.77, adj *p*-value < 0.0001, according to Tukey correction). Regarding unaccusatives, statistical differences between groups were also found in both preverbal (*F* = 26.27, *p*-value < 0.0001) and postverbal subject conditions (*F* = 8.18, *p*-value = 0.0010). The significant differences were detected in both intermediate-control (preverbal: *t* = −5.76, adj *p*-value < 0.0001; postverbal: *t* = 2.98, adj *p*-value = 0.0131, according to Tukey correction) and advanced-control comparisons (preverbal: *t* = 6.25, adj *p*-value < 0.0001; postverbal: *t* = −3.92, adj *p*-value = 0.0009, according to Tukey correction).

To examine the interaction of *Verb Class* (unergative and unaccusative) and *Group* (intermediate, advanced, and control) in informational contexts, a GLMM was applied. In the SV condition, there were no significant differences regarding *Verb Class* and there was no interaction of *Verb Class* and *Group* in the acceptance values. In the VS condition, the differences were significant with respect to *Verb Class* (*F* = 5.45, *p*-value = 0.0244), and there was no interaction of *Verb Class* and *Group*. In the *post hoc* test, there were no significant differences between unergatives and unaccusatives in neither condition for the three groups.

## Discussion and Conclusions

In this study, we have examined the distribution of subjects in the judgments of L1 Greek intermediate and advanced learners of L2 Spanish in two contextualized acceptability tasks.

**FIGURE 4:**
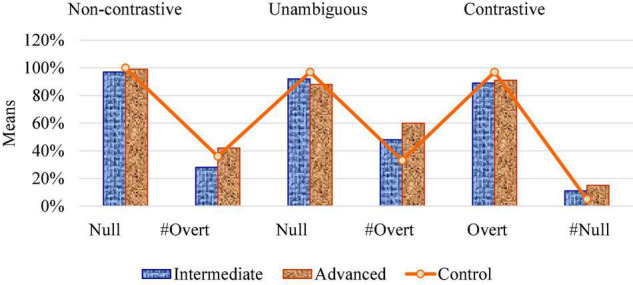
Subject uses in referential contexts.

In Experiment 1, both intermediate and advanced learners showed native-like acceptance of the felicitous null subjects against the unfelicitous overt subjects in non-contrastive referential contexts (Subtest 1). Their performance was independent of the type of person, 1st or 2nd in the null subject condition. However, in the overt subject condition, person had an effect on subject expression, but this was not related to the factor of group, as both L2 groups did not significantly diverge from native patterns. In unambiguous referent-shift contexts (Subtest 2), the L2 groups also showed a higher acceptance of 3rd person null subjects than overt subjects, though they did not reach the ceiling rates of 1st/2nd person in the previous contexts. The intermediate group showed a tendency toward target-like patterns with both null and overt subjects, while the advanced group presented significant divergence from native rates in the case of the unfelicitous overt subjects. In contrastive referent-shift contexts (Subtest 3), the L2 groups followed native-like judgments with both felicitous overt subjects and unfelicitous null subjects of 3rd person. In this case, all groups clearly rejected null pronouns in favor of expressing overt pronouns with contrastive interpretation. The tendencies of the intermediate and advanced groups with respect to the control group are shown in Figure 4 for the three contexts examined in Experiment 1.

**FIGURE 5:**
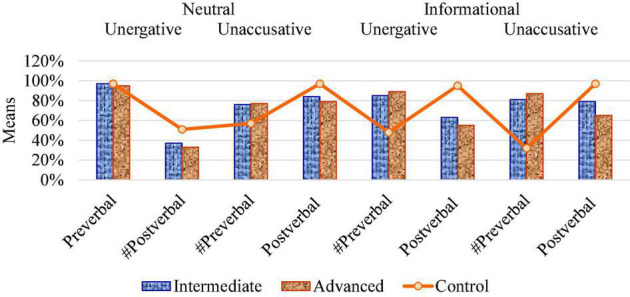
Subject positions in pragmatic contexts.

In Experiment 2, the intermediate and advanced groups showed target-like acceptance of felicitous preverbal subjects with unergative verbs in neutral contexts, but diverged from native-like subject inversion with unaccusative verbs in neutral contexts (Subtest 1). Thus, verb class played a significant role in the judgments of L2 learners with respect to subject position. In informational contexts (Subtest 2), both L2 groups showed divergence from native-like distribution of subjects with unergative and unaccusative verbs. Both groups accepted unfelicitous preverbal subjects, compared to the control group that showed a clear preference for the discursive VS order. In this case, context type had a higher effect on learners’ performance than verb type. See Figure 5 for the word order patterns of the L2 groups with respect to the control group in neutral and informational contexts in Experiment 2.

If we examine the overall results of the two experiments against the predictions formulated in section “Predictions,” we observe that the intermediate and advanced learners of Spanish had no persistent problems with the distribution of null/overt subjects in non-contrastive and contrastive referent-shift contexts in Experiment 1, so that their performance runs against the predictions of the IH-1 (Sorace and Filiaci, 2006) and the IH-2 (Tsimpli and Sorace, 2006). Lozano’s (2016) PPVH can also be rejected in non-contrastive contexts, as the L2 groups did not show significant differences from natives with overt pronouns. However, the advanced group diverged from native rejection of overt subjects in unambiguous referent-shift contexts, showing optionality in their judgments along the predictions of the IH-1/IH-2 and the PPVH.

Regarding Experiment 2, the results run against the IH-2 (Tsimpli and Sorace, 2006), as both L2 groups had difficulties with the distribution of postverbal subjects-unaccusative verbs at the syntax-semantics interface. The syntactic-lexical/semantic properties were not always acquired, and verb class played a role in neutral contexts, as the position of subjects with unergative verbs was acquired earlier by L2 learners. In informational contexts, the non-target performance of both L2 groups showed that they had not yet acquired the syntactic-pragmatic properties of subject distribution. Here the influence of the L1 Greek that allows SV in informational contexts against VS in Spanish (Roussou and Tsimpli, 2006) might be the source of non-target performance, so that the L2 learners overgeneralized the L1 felicitous option.

The results showed that both L2 groups performed better than expected under the IH-1 and IH-2 in non-contrastive and contrastive contexts in Experiment 1. The involvement of pragmatics did not necessarily lead to unacceptable use of subjects in referential contexts. The predictions of Lozano’s (2016) PPVH were not correct in contexts where a null subject was the felicitous option, and the distribution of redundant overt pronouns was variable because they were accepted in unambiguous referent-shift contexts, but rejected in non-contrastive contexts, while ambiguous null subjects were correctly highly avoided in contrastive contexts. Regarding Experiment 2, the predictions of the IH-2 were not fulfilled in the performance of L2 groups. The syntax-semantics interface was not necessarily acquired earlier than the syntax-pragmatics interface. The L2 groups showed a better performance in the case of referential null/overt subjects than in the case of informational preverbal/postverbal subjects, to the effect that not all syntactic-pragmatic properties were equally acquirable or inacquirable in L2, against both versions of the IH.

Overall, the IH-1 and IH-2 failed to account for the results of the two experimental tasks. The PPVH was not fulfilled either in Experiment 1 (the only experiment here for which it made any predictions). The IH-1 did not capture the performance of the intermediate and advanced learners of Spanish in the case of null/overt subjects, but only for the advanced group in unambiguous referent-shift contexts, and for both L2 groups in informational subject-focused contexts. The IH-2 fared well for both L2 groups only in the case of the unergative word order (not for unaccusatives) –not in the case of referential uses of null/overt subjects. The PPVH fared better than the IH in the case of contrastive contexts, but not for non-contrastive contexts. Thus, none of the hypotheses considered captured the performance of L2 learners in all cases examined. Our interpretation would be that the performance of L2ers was affected by grammatical factors, such as the type of subject, verb class and context, and best accounted for in terms of transfer effects. The involvement of interfaces was orthogonal to performance in L2 Spanish by L1 Greek speakers, a result that questions the empirical adequacy of the IH.

## Data Availability Statement

The raw data supporting the conclusions of this article will be made available by the authors, without undue reservation.

## Ethics Statement

The studies involving human participants were reviewed and approved by the Comissió d’Ètica en l’Experimentació Animal i Humana (CEEAH), Universitat Autònoma de Barcelona. The participants provided their written informed consent to participate in this study.

## Author Contributions

PM and AG designed this study. PM conducted the experiments and statistical analysis. Both authors are responsible for interpreting the results, and writing the manuscript.

## Conflict of Interest

The authors declare that the research was conducted in the absence of any commercial or financial relationships that could be construed as a potential conflict of interest.

## Publisher’s Note

All claims expressed in this article are solely those of the authors and do not necessarily represent those of their affiliated organizations, or those of the publisher, the editors and the reviewers. Any product that may be evaluated in this article, or claim that may be made by its manufacturer, is not guaranteed or endorsed by the publisher.

## Footnotes

1 Sorace and Serratrice (2009: 200) proposed an extension of the Interface Hypothesis (IH-3) for bilingual acquisition, in the following terms: “Processing limitations in bilingual speakers may be responsible at least for some of the difficulties attested at the interfaces, especially the ones requiring the coordination of syntactic and contextual information, while internal interfaces may be expected to be less sensitive to processing limitations because they involve mappings between formal properties of the language system alone.” This is an extension of the IH to bilingual populations, which are not the focus of this manuscript.

2 Here we take into consideration White’s (2011a,b) claim that the IH should hold of developmental stages and we examine not only the very advanced levels (“the near-natives” as originally proposed by the IH), but also the intermediate levels of knowledge, which are extensively studied in the literature for the interface framework (Lozano, 2006a,b, 2018; Domínguez and Arche, 2014).

3 A reviewer suggests that a between-subjects design would have been more appropriate, with several lists and the distribution of the experimental items per list following the Latin Square procedure, and would have allowed for more distractors, something to take into account for future research.
